# Inkjet-Printed Molybdenum Disulfide and Nitrogen-Doped Graphene Active Layer High On/Off Ratio Transistors

**DOI:** 10.3390/molecules25051081

**Published:** 2020-02-28

**Authors:** Mohi Uddin Jewel, Mahmuda Akter Monne, Bhagyashree Mishra, Maggie Yihong Chen

**Affiliations:** 1 Ingram School of Engineering, Texas State University, San Marcos, TX 78666, USA; jewel.eee08@gmail.com; 2 Materials Science, Engineering, and Commercialization, Texas State University, San Marcos, TX 78666, USA; mahmuda.a.monne@txstate.edu (M.A.M.); b_m415@txstate.edu (B.M.)

**Keywords:** inkjet printing, graphene, molybdenum disulfide, Raman, thin-films, cross-section, nanosheets, on/off ratio, transistor

## Abstract

Fully inkjet-printed device fabrication is a crucial goal to enable large-area printed electronics. The limited number of two-dimensional (2D) material inks, the bottom-gated structures, and the low current on/off ratio of thin-film transistors (TFTs) has impeded the practical applications of the printed 2D material TFTs. In the search for TFTs with high current ratios, we introduce a stable and efficient method of nitrogen-doped graphene (NDG) ink preparation for inkjet printing by liquid-phase exfoliation. The NDG thin film is print-stacked with molybdenum disulfide (MoS_2_) by multiple printing passes to construct a MoS_2_–NDG stack. We demonstrate top-gated fully inkjet-printed MoS_2_–NDG transistors with silver drain, source, and gate electrodes, and a barium titanate (BaTiO_3_) dielectric. A 100% inkjet-printed MoS_2_–NDG vertical 2D active heterostructure layer transistor with a current on/off ratio of 1200 is exhibited. The results may lead towards the development of all-printed 2D material-based transistor switches.

## 1. Introduction

Graphene and related two-dimensional (2D) materials remain at the center of research for more than a decade because of their exciting and unusual properties [1]. Printing of solution-processed 2D materials facilitates the fabrication of the next generation of flexible devices. Inkjet printing as a process for flexible and printed electronics, has become more popular for the fabrication of electronic devices because of its ability for large-area fabrication, the limited number of process steps involved, low-temperature processing, and low cost [2]. Graphene is a zero-band gap semimetal in which the valence and conduction bands are cone shaped, and they meet at the K point of the Brillouin zone. Due to the absence of band gap, the typical current on/off ratio of pristine graphene thin-film transistors (TFTs) is approximately 5 [3]. For switching applications of TFTs, a current on/off ratio between 10^4^ and 10^7^ and a band gap of thin film larger than 0.36 eV are desirable [4]. As with regular thin-film graphene TFTs, the inkjet-printed graphene transistors had current ratios of ≈10 [2,5]. Recently, all inkjet-printed graphene transistors were reported for wearable and textile electronics [6]. However, the current on/off ratio was still small (about 2.5). Molybdenum disulfide (MoS_2_), a semiconducting layered transition metal dichalcogenide (TMD), is one of the most studied 2D materials after graphene. Mono and few-layered MoS_2_ have a direct band gap of 1.8 eV, while bulk MoS_2_ has an indirect band gap of 1.29 eV [7]. Despite having a band gap in MoS_2_, liquid phase-exfoliated (LPE) and inkjet-printed MoS_2_ transistors had low current on/off ratios (<10) [8,9,10,11]. A maximum current on/off ratio of 600 was demonstrated for the electrolytically gated TFTs based on porous nanosheet networks (PNNs) of 2D materials [12]. However, the TFTs were not fully printed. For example, the channel materials were spray coated, the source and drain electrodes were deposited by electron beam (e-beam) evaporation, and the liquid electrolyte was drop casted [12]. Yet, their reported all-printed TFTs with inkjet-printed graphene source, drain, and gate, and tungsten diselenide (WSe_2_) channel, a spray-coated porous boron nitride (BN) electrochemical separator (not a dielectric), and a drop-casted ionic liquid, displayed on/off ratios of ≈25. Very recently, solution-processed 2D material back-gated TFTs with an on/off ratio of >10^5^ were reported using standard photolithography process [13]. The devices available in the literature are mostly bottom gated and printed devices have low on/off ratios which are not suitable for practical application of a transistor switch. Therefore, it is indispensable to develop top-gated TFTs with high on/off ratios for printed transistor switches.

Several attempts have been made to open the band gap in graphene through graphene–substrate interaction, chemical substitution doping, and quantum confinement [14,15,16]. A band gap of 0.25 eV was achieved for chemical vapor-deposited (CVD) nitrogen-doped graphene (NDG) nanosheets [15]. Because of the band gap opening in NDG, it has a great potential to be an excellent material for inkjet-printed transistors with high current ratios when coupled with semiconducting MoS_2_. In this work, we report the development of NDG and MoS_2_ inks, fabrication, and characterization of a fully printed top-gated MoS_2_–NDG stack field effect transistor. We designed a printed vertical MoS_2_–NDG stack for the transistor channel. The all-printed transistor displays a current on/off ratio of 1200.

## 2. Results

### 2.1. NDG and MoS_2_ Nanosheets

**Figure 1:**
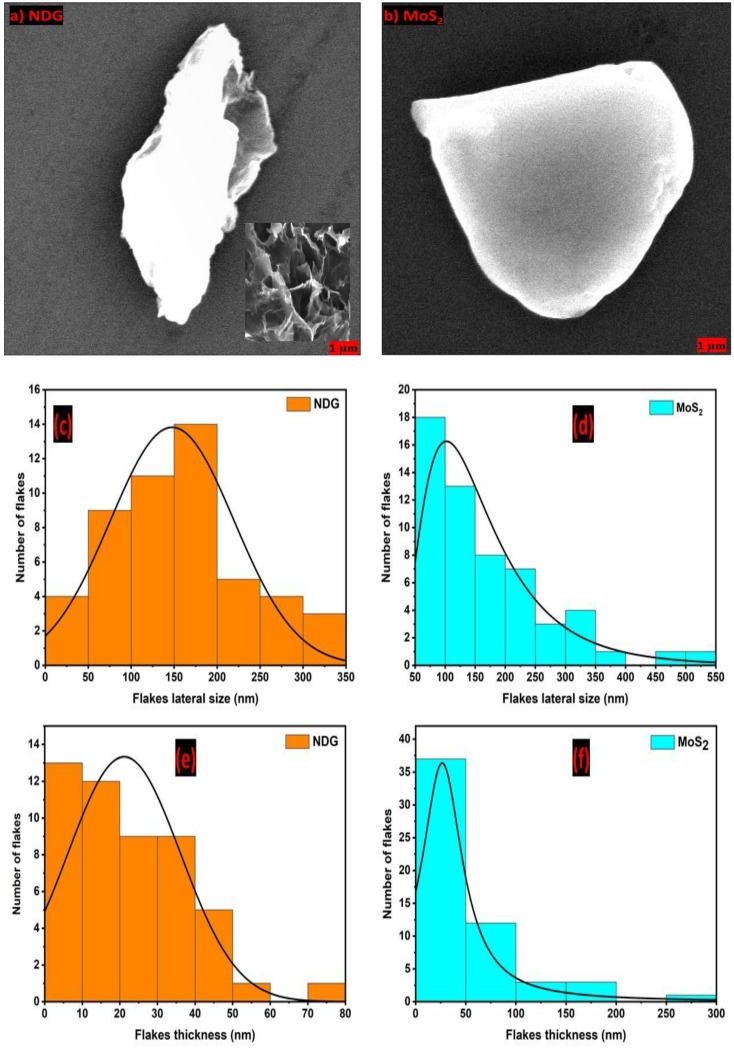
Scanning electron microscopy (SEM) images of (**a**) bulk nitrogen-doped graphene (NDG) nanosheets (inset: SEM image of broken NDG nanosheets), and (**b**) molybdenum disulfide (MoS_2_) as-purchased flakes. Lateral size distribution from atomic force microscopy (AFM) measurements of (**c**) NDG and (**d**) MoS_2_ flakes. Flake thickness distribution of (**e**) NDG and (**f**) MoS_2_.

The flake dimensions must match the requirements of drop-on-demand (DOD) printing. The lateral dimensions of the dispersed nanosheets should be at least 1/50 of the nozzle diameter (a ≈ 21.5 µm) to prevent the nozzle clogging. We purchased the NDG powder from Sigma-Aldrich (product no. 900527) and MoS_2_ ultrafine powder from Graphene supermarket. The scanning electron microscopy (SEM) images of bulk nanosheets are shown in Figure 1a–b. Prior to dispersing the nanosheets into solvents, the nanosheets were sonicated for 10 h to break the bulk nanosheets. The lateral size and thickness of the individual NDG and MoS_2_ nanosheets were extracted from atomic force microscopy (AFM) measurements for over 50 flakes as shown in Figure 1c–f. The flake size follows the normal distribution (peak ≈ 150 nm) for NDG and log-normal distribution (peak ≈ 100 nm) for MoS_2_, respectively, which fulfills the DOD requirements. The statistical thickness of normal distribution for NDG nanosheets peaked at 20 nm, and the Lorentz distribution of thickness for MoS_2_ nanosheets peaked at 25 nm, respectively. Assuming a thickness of 0.37 nm for monolayer graphene and 0.615 nm for monolayer MoS_2_ [17,18], the nanosheets consist of an average number of layers; N ≈ 54 for the NDG and N ≈ 40 for the MoS_2_.

**Figure 2:**
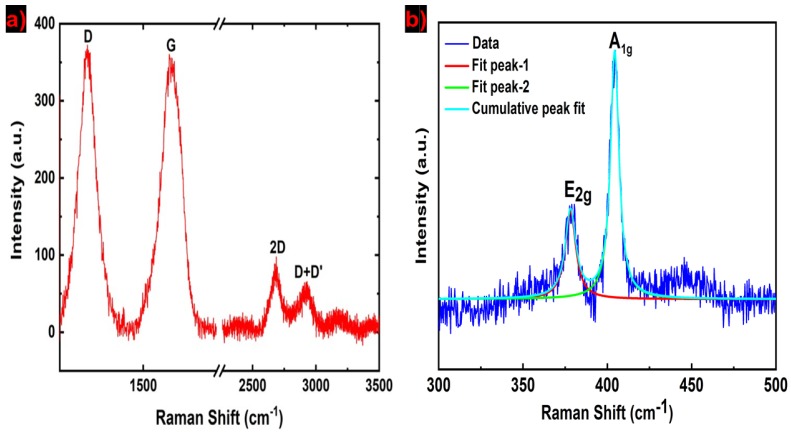
(**a**) Raman spectrum of NDG nanosheets; (**b**) Raman spectrum and their Lorentzian fits of MoS_2_ nanosheets.

Raman is one of the most common vibrational spectroscopies for fingerprinting carbon species. After the sonication, the Raman spectrum of NDG nanosheets on Si substrate was acquired under ambient conditions, which is shown in Figure 2a. The NDG shows two intense peaks, which are the D (≈1341 cm^−1^) and G (≈1578 cm^−1^) peaks, respectively. There are two additional peaks, 2D (≈2670 cm^−1^) and D + D’ (≈2904 cm^−1^), present in the NDG nanosheets. The G and 2D peaks are the signature of graphene-like structure in nanosheets. The G band corresponds to E_2g_ phonons at the Brillouin zone center. The G band arises from the stretching of C–C bonds in a sp^2^ hybridized carbon system. The D band is related to defects in sp^2^ hybridized carbon materials, which is activated by an inter-valley double resonance (DR) Raman process. The intensity ratio I_D_ (intensity of D peak)/I_G_ (intensity of G peak) of 1.005 indicates a high degree of disorder due to nitrogen doping. The strong D peak can also originate from the submicrometer flake boundaries and vacancies. The 2D peak is the secondary D peak, which appears at approximately 2700 cm^−1^. For single-layer graphene, the 2D band shows a sharp peak and high intensity compared to the D and G peaks [19]. The 2D band broadens and the intensity reduces with the number of graphene layers. In the Raman spectrum of Figure 2a, the intensity of the 2D band is much smaller compared to the D and G bands. We attribute the low intensity of the 2D band to the presence of many layers (N ≈ 54) in NDG nanosheets. This Raman spectrum strongly agrees with the Raman spectra previously reported for NDG synthesized by chemical vapor deposition at 800 and 900 °C [20]. Figure 2b shows the Raman spectrum of MoS_2_ flakes with characteristic E_1g_ and A_1g_ peaks at 378 and 405 cm^−1^ respectively, which are obtained by Lorentzian fits in the spectrum.

### 2.2. Printable Inks and Thin Films

In this study, we used a commercial drop-on-demand (DOD) Fujifilm Dimatix materials printer (DMP-2800). For DOD inkjet printing, the material inks must have the ability to generate droplets. The droplets ejecting out from a nozzle are influenced by ink viscosity η (mPa s), surface tension γ (mN/m), particle density ρ (g/cm^3^), and nozzle diameter a (µm). We prepared and characterized the NDG and MoS_2_ inks (see ’Materials and Methods’). For inkjet printing of an ink, the inverse Ohnesorge number (Z = Oh^−1^) is used as the figure of merit (FOM) to characterize the drop formation and jettability of an ink. Z is calculated by using Z = (γρa)1/2η and an optimal range of Z between 1 and 14 is required for stable DOD printing [5]. For NDG ink, using η = 2.94 mPa s, γ = 20.98 mN/m, ρ = 1.054 g/cm^−3^, a = 21.5 µm, we obtain Z = 7.5, which is within the conventional range for inkjet printing. There were no satellite drops following the primary drops, which was confirmed by capturing the dynamics of drop formation. The Z for MoS_2_ and barium titanate (BaTiO_3_) inks were 2 and 3.4, which are suitable for inkjet printing.

**Figure 3:**
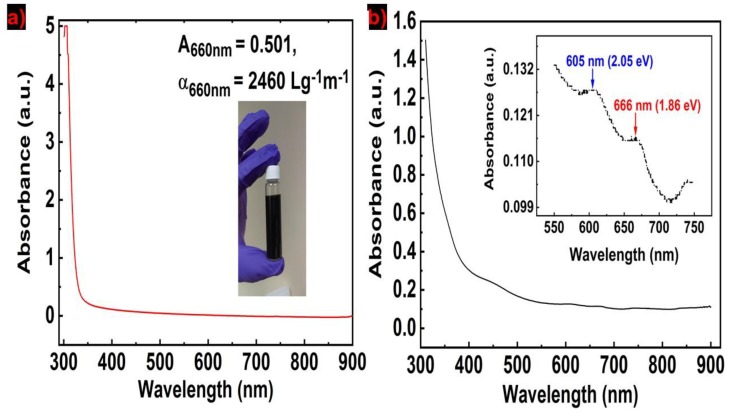
(**a**) Optical absorption of NDG ink shown in the inset; (**b**) absorbance spectra of MoS_2_ ink (inset: peak positions in MoS_2_ ink).

The concentration of nanosheets in the ink can be calculated from the UV–vis absorbance spectrum via the Beer–Lambert law. The Beer–Lambert law can be expressed as A = αcl, where A is the absorbance, α is the absorption coefficient (Lg^−1^m^−1^), c is the concentration of dispersed graphene (g/L), and l is the light path length (m). The optical absorption of the NDG ink is shown in Figure 3a. The spectrum is mostly featureless due to the linear dispersion of the Dirac electrons, whereas the peak in the UV region is a signature of Hove singularity in the graphene density of states [6]. From A = 0.501, α = 2460 Lg^−1^m^−1^, l = 0.01 m, our estimated NDG concentration is 0.4 mg/mL [21]. Figure 3b shows the optical absorbance spectra for as prepared MoS_2_ ink. This absorbance spectrum complies with the previously reported MoS_2_ ink absorbance spectra [8]. For MoS_2_ presence in dispersion, two characteristic peaks appear at approximately 600 and 672 nm wavelength respectively [10]. Two excitonic absorption peaks at 605 and 666 nm are observed, which arise from the K points of the Brillouin zone in MoS_2_ nanosheets. The peak at 666 nm corresponds to the lowest optical bandgap of 1.86 eV for MoS_2_ nanosheets, which is higher than the bulk MoS_2_ bandgap of 1.3 eV [22]. The extinction coefficient of MoS_2_ at 672 nm wavelength is ~α_672_ = 3400 mL/(mg.m) [8]. Using the Beer–Lambert law, the calculated final MoS_2_ concentration is ~0.1 mg/mL.

**Figure 4:**
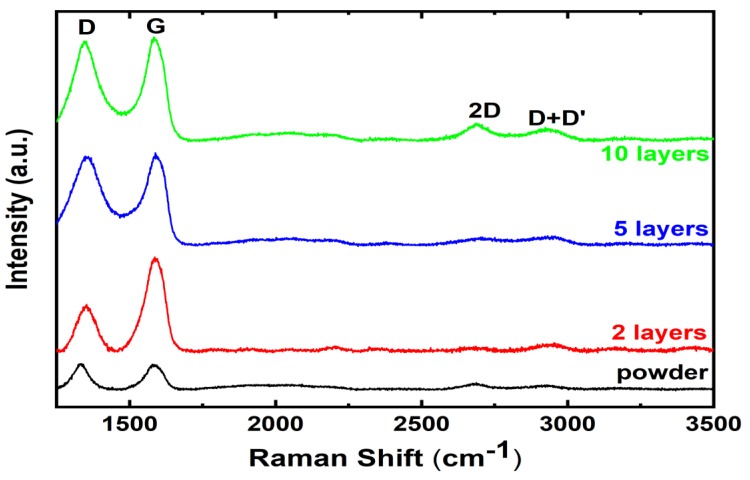
Raman spectra of printed NDG flakes compared with NDG powder.

Figure 4 compares the Raman spectrum of the NDG powder and printed layers. As with NDG powder, the Raman spectra of the printed thin films have D, G, 2D, and D + D’ peaks, respectively. The peaks of the Raman spectra remain identical with additional printing passes. The distribution of Raman bands in the printed layers remain similar to the NDG powder. The lower intensity of 2D peak indicates the presence of many layers of graphene in the printed thin films. Doping has strong effects on the position and FWHM of the 2D peak. The 2D peak position varied between 2667 and 2711 cm^−1^. We also calculated the full width at half maximum (FWHM) of the 2D peaks for the Raman spectra shown in Figure 4. The FWHM for the NDG powder, 2, 5 and 10 printed layers was 77.4, 63.6, 62.6, and 92.9, respectively. This, along with 2D peak position shift, implies that the nitrogen doping has a strong effect on printed NDG samples.

### 2.3. MoS_2_–NDG Stack

**Figure 5:**
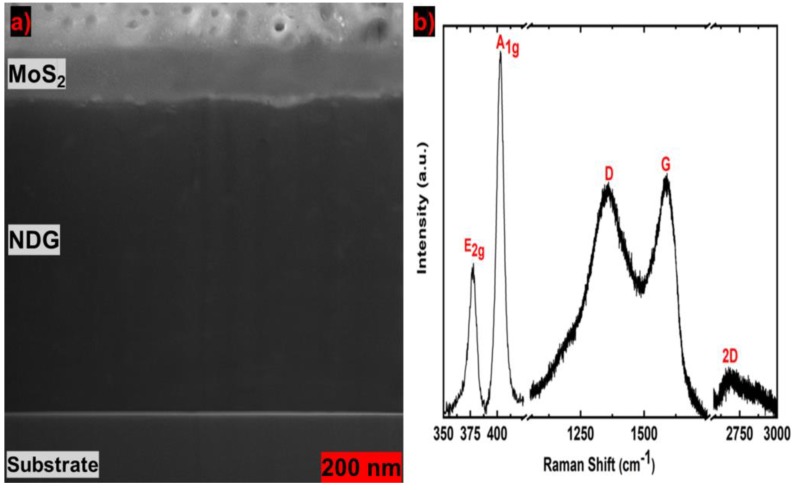
(**a**) FIB-SEM cross-sectional image of the MoS_2_–NDG stack, and (**b**) Raman spectrum of the printed MoS_2_–NDG stack on a glass substrate.

The 2D materials can be arranged into a stack to create a materials assembly with novel properties [23]. In the stack, atomically thin 2D materials are bonded by weak van der Walls forces [24]. The MoS_2_ dispersions produced by LPE have the optical bandgap ranges from 1.8 eV to 1.3 eV [12]. We created and optimized the stack structures of printed MoS_2_ and NDG materials. To inspect the inside of a stack, a cross-sectional slice was produced by focused ion beam (FIB) milling for a thicker sample. Figure 5a shows the FIB-SEM cross-sectional image of the MoS_2_–NDG stack. The cross-sectional thickness of the NDG and MoS_2_ prints are 527 and 105 nm, respectively. The interfacial separation between the MoS_2_ and NDG is not abrupt and well-maintained along the cross-section. There is no identifiable large flake in the cross-section. This ensures that the nanosheets are dissolved well in the solvents, and our sonication and centrifugation processes are reliable in removing thicker flakes. Raman of the MoS_2_–NDG stack in Figure 5b shows the characteristic MoS_2_ peaks (E_2g_ ~ 378 cm^−1^, A_1g_ ~ 403 cm^−1^) and NDG peaks (D ~ 1357 cm^−1^, G ~ 1590 cm^−1^, and 2D ~ 2681 cm^−1^).

### 2.4. Transistor Fabrication and Characterization

**Figure 6:**
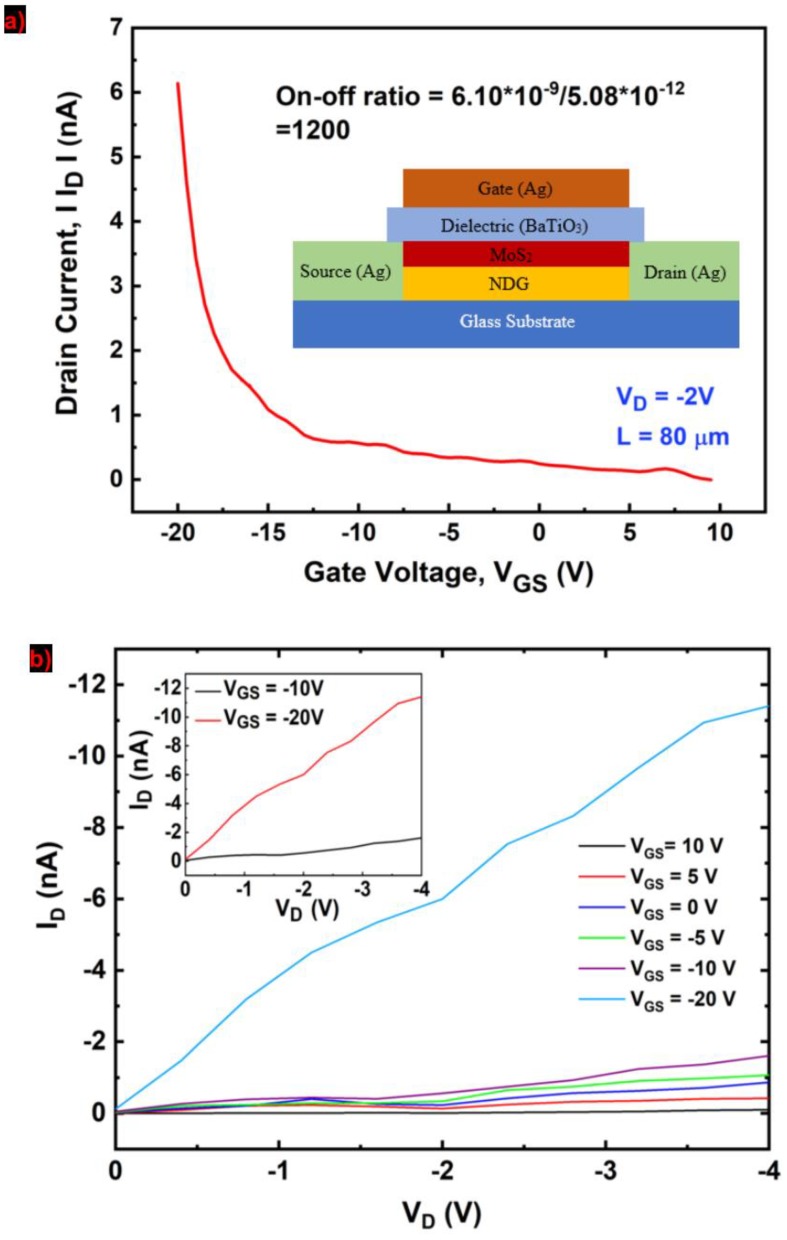
(**a**) Transfer curve of the MoS_2_–NDG transistor shown in the inset, and (**b**) output characteristics of the same device.

The transistors themselves are all inkjet printed on the glass substrate without involving any photolithography patterning or surface pretreatment steps. The TFTs are top gated, consisting of the source, drain, gate electrodes, a channel, and a dielectric layer. The inset of Figure 6a shows the schematic structure of an NDG transistor. Highly conductive silver nanoparticle ink (Novacentrix Metalon^®^ JS-B40G) was used to print source, drain and gate terminals. As the very first layer, source and drain electrodes were printed on the glass substrate in a printing pass. The printed silver patterns were cured at 150 °C for 30 min. The separation between source and drain electrodes, i.e., the channel length (L) was ~80 µm. The ed silver contacts deposited uniformly with an average thickness of 0.50 µm. For channel region, a percolation network of 100 nm NDG was printed with 40 printing passes followed by a MoS_2_ printing repeated 4 times on top of NDG. The channel layer had some overlapping with source and drain contacts to avoid discontinuity in the structure. Barium titanate (BaTiO_3_) ink (*k* ~ 20.5) of a thickness of ~2 µm was used as the dielectric (see ‘Materials and Methods’). The printed dielectric was first baked at 100 °C for 20 min, and subsequently baked at 230 °C for 30 min. Finally, the silver gate electrode was printed on top of the dielectric layer.

Figure 6a shows the non-linear transfer curve of the transistor at V_D_ = −2 V. The applied gate voltage changed from −20 to 10 V. The I_D_ increases as the gate voltage changes from positive to negative, which suggests that the stack creates a p-type transport layer. A current on/off ratio of 1200 is calculated at V_D_ = −2 V from the transfer characteristics. The electric field mobility ($\mu \;=\;\frac{L\times g_{m}}{W\times C_{\mathrm{ox}}\times V_{D}}$) is calculated under the assumption of constant electric field along the channel and no velocity saturation. The device mobility was ~0.01 cm^2^/V.s, which is comparable with liquid-exfoliated MoS_2_–based TFTs [8]. Moreover, the device has a good off state. We measured the gate leakage (I_G_) from −10 to +10 V. The I_G_ shows random signal in this range and does not indicate any gate leakage and the drain current (I_D_) was independent of the gate current (Supplementary Information). Figure 6b shows the output characteristics of the device at a gate voltage sweep from 10 to −20 V. There is no Schottky barrier and the current started flowing upon applying V_D_ implying the contact is ohmic and is the result of direct tunneling process. The transfer and output characteristics of the device exhibit p-type behaviors. To benchmark the on/off ratio results, we also investigated the inkjet-printed NDG transistors. The similar fabrication procedures were carried out for NDG transistors (L ~ 80 µm) on the glass substrate with same Ag electrodes and BaTiO_3_ gate dielectric. For NDG transistors, a channel of 100 nm thickness was created with 40 printing repetitions to ensure the availability of NDG nanosheets on the top surface of the NDG thin films. The channel layer had some overlapping with source and drain contacts to avoid discontinuity in the structure. Transfer characteristics of the NDG transistor are shown in Figure S4a. The gate voltages were changed from −20 to +20 V. The transfer characteristics were measured at V_D_ = 5, 10, 15, and 20 V, respectively. The drain current increased as the V_GS_ changed from negative to positive, which confirmed the n-type doping in NDG nanosheets due to nitrogen doping. However, the drain current did not change significantly over a wide range of gate bias. For example, at V_D_ = 20 V, the drain current changed from 1105 to 1247 mA in the given gate voltage range. The current on/off ratio can be calculated from the transfer characteristics and it is the ratio of maximum to minimum drain current for a specific V_D_. At V_D_ = 20, the on/off ratio of the NDG is ~1.13. This on/off ratio is comparable to other inkjet-printed graphene TFTs, but not suitable for transistor switches. The drain currents were measured in V_GS_ = −20, −10, 0, 10, and 20 V, respectively. Still the linear I_D_–V_D_ curves were not distinguishable for various gate voltages, i.e., the field effect in the output curves were not apparent. A linear I_D_–V_D_ curve for V_GS_ = 10 V is displayed in Figure S4b as the output characteristics. We also fabricated several MoS_2_ transistors following same fabrication processes for a channel length of ~80 µm. However, we were unsuccessful in achieving a working transistor. Our interpretation in this matter is that the printed MoS_2_ layers failed to form a semiconducting percolation network on a bare glass substrate. However, MoS_2_ layers when printed on top of NDG layers, formed a continuous MoS_2_ percolation network and maintained a clear separation from the NDG layers underneath due to hydrophobic nature of NDG surface. Some MoS_2_ flakes may penetrate to the NDG layers, which reduced the overall conductivity of the NDG channel. Therefore, the high current on/off ratio originated from printed MoS_2_ and the NDG layers underneath supported MoS_2_ to start the nucleation when printed.

## 3. Discussion

We demonstrated a high current on/off ratio transistor based on the MoS_2_–NDG stack as the channel. The inks, thin films, and devices were characterized by various experimental characterization techniques. The NDG transistor was used as a control structure which showed low on/off ratios. Upon printing MoS_2_ on top of NDG to create a MoS_2_-NDG stack, semiconducting properties were achieved, and on/off ratios increased significantly for a channel length of 80 µm.

## 4. Materials and Methods

**Nitrogen-doped graphene (NDG) ink formulation.** The NDG ink was prepared by dispersing NDG flakes into terpineol/cyclohexanone mixture. The NDG powder (≈5 mg, Sigma-Aldrich product no. 900527, Saint Louis, MO 63103, USA) was sonicated for 10 h to break the bigger particles. For a 10 mL solution, 1.5 mL of terpineol was used as the solvent. To curtail the viscosity of terpineol, 8.5 mL of cyclohexanone was added. Then, ethyl cellulose (as a surfactant) of 48 mg was mixed into the solution and sonicated for 1 h. Finally, the sonicated NDG powder (≈5 mg) was poured into the solution. The resulting suspension was further sonicated for 20 h, and then centrifuged at 5000 rpm for 30 min to remove the thicker flakes. After centrifugation, the suspension was left to settle overnight. The final ink was filtered out from the residue and ready for characterization.

**Nitrogen-doped graphene (NDG) ink characterization.** The surface tension of NDG ink was measured using the pendant drop method (First Ten Angstroms FTA10000B, Portsmouth, VA 23704, USA). A small amount of ink (3.8 µL) was suspended from a needle and surface tension was extracted from the software image. The ink density is ρ = m/V, m is the mass of ink in grams and V is volume of ink in mL. The ink density ρ for NDG ink was 1.054 g/cm^3^. The viscosity of the ink was measured with the RheoSense m-VROC viscometer. The viscosity of NDG ink was ≈2.94 mPa s. Similar characterization was carried out for the MoS_2_ ink.

**Atomic force microscopy (AFM).** A Bruker atomic force microscope (Billerica, MA 01821, USA) was used to extract the flake thickness and lateral distribution. The flakes were scraped on a silicon substrate after sonication before mixing with solvents. The scan areas were 500 × 500 µm^2^ and 200 × 200 µm^2^ for the nanosheets.

**Scanning electron microscopy.** Scanning electron microscopy was carried out using a Helios Nanolab 400 FEI SEM (Hillsboro, OR 97124, USA). The field emission gun was operated at an accelerating voltage of 5 kV and gun current of 0.34 nA. The images were captured in the field-free mode of SEM using TLD detector at the indicated magnification scale in SEM images.

**Raman spectroscopy.** Raman measurements were performed on the sonicated bulk NDG powder and inkjet-printed layers using a Horiba LabRAM HR evolution Raman spectrometer (Austin, TX 78754, USA). The laser excitation of 532 nm was used at 100 × objective, with an incident power less than 1 mW to avoid sample damage.

**Molybdenum disulfide (MoS_2_) ink.** The MoS_2_ ultrafine powder was purchased from Graphene Supermarket (Ronkonkoma, NY 11779, USA). The NMP (1-Methyl-2-pyrrolidinone) solvent was diluted to DI water by a 45:55 mL ratio. The weight of NMP/DI water composition was measured and mixed with 2% weight equivalent of MoS_2_ powder. The mixture was sonicated for 20 h and followed by a centrifugation at 5000 rpm for 30 min. The supernatant was filtered out from the top of the mixture after it rested overnight. The resultant MoS_2_ ink was yellowish. The ink viscosity, surface tension, and density were 3.8 mPa s, 25 mN/m, and 0.1 g/cm^3^, respectively.

**Optical absorption spectroscopy.** UV–vis absorption spectroscopy was used to calculate the flakes concentration in NDG and MoS_2_ inks utilizing the Beer–Lambert law. For the absorbance spectra measurements, the NDG ink was diluted in isopropyl alcohol (IPA) at a ratio of 1:20 and the MoS_2_ ink was as prepared. The measurements were performed with a Shimadzu UV-2501 spectrophotometer (Kyoto, Japan) using a glass cuvette with an optical path length of 10 mm. For concentration calculation, absorption coefficients of *α*_GR_ ~ 2460 for the graphene ink at 660 nm wavelength [21], and *α*_MoS2_ ~ 3400 for the MoS_2_ ink [8] at 672 nm wavelength were used.

**Barium titanate (BaTiO_3_) ink preparation.** The dielectric ink includes 20% by weight of BaTiO_3_ nanoparticles, 1% by weight of the Triton X-100, 14.5% by weight of Cyclopentanone, and 64.5% by weight of SU8 2005 photoresist. For ink preparation, SU8 2005 (MicroChem, Newton, MA 02464, USA), Cyclopen-tanone (Sigma-Aldrich, product—W391018), and Triton X-100 (Sigma-Aldrich, product—X100) were mixed and sonicated for 4 h. The BaTiO_3_ nanoparticles (US Research Nanomaterials, product—US3827, Houston, TX 77084, USA) are then added to the mixture, and the composition was sonicated for an additional 6 h. The resultant mixture was left untouched for one day to form a stable dispersion. The ink was collected from the suspension, leaving the residue of thick powders at the bottom of the bottle. The density of BaTiO_3_ was measured to be 1.4 g/cm^3^. The surface tension of 55 mN/m and viscosity of 12 mPa s were considered for inverse Ohnesorge number calculation [25]. The effective dielectric constant (*k*) of the printed dielectric was measured from the transmission coefficient S_12_ with a T-shape microstrip line resonator. The effective dielectric constant (*k*) was ~20.5. The *k*-value of printed dielectric is consistent with the previous report [26].

**Viscosity measurement.** The viscosity was measured at room temperature with a Rheosense m-VROC viscometer (San Ramon, CA 94583, USA). In m-VROC Rheosense software (version: 3.1.4), we entered the sample size in mL and an estimated viscosity for an ink and it automatically calculated minimum and maximum flow rate. We set five different flow rates within the displayed range and measurement times and dispersed the sample through the measurement sensor. The regression coefficient (R^2^) was greater than 0.99 for all our viscosity measurements

**Inkjet Printing.** A DoD Fujifilm Dimatix (DMP-2800, Santa Clara, CA 95050, USA) inkjet printer was used to print all the thin films and devices. It was equipped with a Dimatix materials cartridge (DMC-11610). The cartridge head has 16 piezoelectric jetting nozzles with a diameter of 21.5 µm each. The nozzles can dispense a droplet of the nominal volume of 10 pL from the cartridge head. The optimum drop spacing for the NDG and MoS2 inks was 30 µm, and six nozzles were used to print these inks. The BaTiO_3_ ink was printed with a 35 µm drop spacing with one nozzle. There was no delay between subsequent printing passes except a 0.3 s purge in every ten printing passes. Additionally, to avoid the coffee ring effect, the platen temperature was set at 60 °C. The 2D device patterns were created in AutoCAD, where the design layout represents different parts of the transistor. The design patterns were converted into a printable version of the Fujifilm Dimatix printer with ACE 300 software.

**Electrical characterization.** The current–voltage measurements of the devices were performed with the Keysight B1500A semiconductor device analyzer.

## 5. Summary

In summary, we developed the NDG and MoS_2_ inks for inkjet printing and studied the inks and thin-film properties through experimental characterizations. We devised a way to prepare a MoS_2_–NDG stack to use as the transistor channel. The devices were fully inkjet-printed without any involvement of Cleanroom facility. Based on the test results of the DC analysis of the device, the current on/off ratio of 1200 is reported in this work.

## Supplementary Materials

The following are available online. Table S1: Data table for Raman measurements; Figure S1: Optical image of final transistor; Figure S2: Absorbance spectrum for solvents of (a) NDG ink, and (b) MoS_2_ ink; Figure S3: Gate leakage measurement of the MoS_2_–NDG transistor; Figure S4: (a) Transfer characteristics, and (b) output curve of the NDG transistor; Table S2: Comparison of on/off ratios of 2D material TFTs; Figure S5: Comparison of current on/off ratios.

## References

[1] Geim A.K., Novoselov K.S. (2007). The rise of graphene. Nat. Mater..

[2] Li J., Ye F., Vaziri S., Muhammed M., Lemme M.C., Östling M. (2013). Efficient inkjet printing of graphene. Adv. Mater..

[3] Xia F., Farmer D.B., Lin Y., Avouris P. (2010). Graphene field-effect transistors with high on/off current ratio and large transport band gap at room temperature. Nano Lett..

[4] Schwierz F. (2010). Graphene transistors. Nat. Nanotechnol..

[5] Torrisi F., Hasan T., Wu W., Sun Z., Lombardo A., Kulmala T.S., Hsieh G.-W., Jung S., Bonaccorso F., Paul P.J. (2012). Inkjet-printed graphene electronics. ACS Nano.

[6] Carey T., Cacovich S., Divitini G., Ren J., Mansouri A., Kim J.M., Wang C., Ducati C., Sordan R., Torrisi F. (2017). Fully inkjet-printed two-dimensional material field-effect heterojunctions for wearable and textile electronics. Nat. Commun..

[7] Li X., Zhu H. (2015). Two-dimensional MoS_2_: Properties, preparation, and applications. J. Mater..

[8] Coleman J.N., Lotya M., O’Neill A., Bergin S.D., King P.J., Khan U., Young K., Gaucher A., De S., Smith R.J. (2011). Two-dimensional nanosheets produced by liquid exfoliation of layered materials. Science.

[9] Lee K., Kim H., Lotya M., Coleman J.N., Kim G., Duesberg G.S. (2011). Electrical characteristics of molybdenum disulfide flakes produced by liquid exfoliation. Adv. Mater..

[10] Li J., Naiini M.M., Vaziri S., Lemme M.C., Östling M. (2014). Inkjet printing of MoS_2_. Adv. Funct. Mater..

[11] He Q., Zeng Z., Yin Z., Li H., Wu S., Huang X., Zhang H. (2012). Fabrication of flexible MoS_2_ thin-film transistor arrays for practical gas-sensing applications. Small.

[12] Kelly A.G., Hallam T., Backes C., Harvey A., Esmaeily A.S., Godwin I., Coelho J., Nicolosi V., Lauth J., Kulkarni A. (2017). All-printed thin-film transistors from networks of liquid-exfoliated nanosheets. Science.

[13] Lin Z., Liu Y., Halim U., Ding M., Liu Y., Wang Y., Jia C., Chen P., Duan X., Wang C. (2018). Solution-processable 2D semiconductors for high-performance large-area electronics. Nature.

[14] Zhou S.Y., Gweon G.-H., Fedorov A.V., First de P.N., De Heer W.A., Lee D.-H., Guinea F., Neto A.H.C., Lanzara A. (2007). Substrate-induced bandgap opening in epitaxial graphene. Nat. Mater..

[15] Lawlor J.A., Ferreira M.S. (2014). Sublattice asymmetry of impurity doping in graphene: A review. Beilstein J. Nanotechnol..

[16] Han M.Y., Özyilmaz B., Zhang Y., Kim P. (2007). Energy bandgap engineering of graphene nanoribbons. Phys. Rev. Lett..

[17] Koh Y.K., Bae M.-H., Cahill D.G., Pop E. (2010). Reliably counting atomic planes of few-layer graphene (n > 4). ACS Nano.

[18] Addou R., Colombo L., Wallace R.M. (2015). Surface defects on natural MoS_2_. ACS Appl. Mater. Interfaces.

[19] Ferrari A.C., Meyer J.C., Scardaci V., Casiraghi C., Lazzeri M., Mauri F., Piscanec S., Jiang D., Novoselov K.S., Roth S. (2006). Raman spectrum of graphene and graphene layers. Phys. Rev. Lett..

[20] Lu Y.-F., Lo S.-T., Lin J.-C., Zhang W., Lu J.-Y., Liu F.-H., Tseng C.-M., Lee Y.-H., Liang C.-T., Li L.-J. (2013). Nitrogen-doped graphene sheets grown by chemical vapor deposition: Synthesis and influence of nitrogen impurities on carrier transport. ACS Nano.

[21] Hernandez Y., Nicolosi V., Lotya M., Blighe F.M., Sun Z., De S., McGovern I.T., Holland B., Byrne M., Gun’Ko Y.K. (2008). High-yield production of graphene by liquid-phase exfoliation of graphite. Nat. Nanotechnol..

[22] Mishra A.K., Lakshmi K.V., Huang L. (2015). Eco-friendly synthesis of metal dichalcogenides nanosheets and their environmental remediation potential driven by visible. Sci. Rep..

[23] Bonaccorso F. Ink-jet printed 2D crystal heterostructures. Proceedings of the 2017 47th European Solid-State Device Research Conference (ESSDERC).

[24] Roy T., Tosun M., Kang J.S., Sachid A.B., Desai S.B., Hettick M., Hu C.C., Javey A. (2014). Field-effect transistors built from all two-dimensional material components. ACS Nano.

[25] Vukmirovic J., Tripkovic D., Bajac B., Kojic S., Stojanovic G., Srdic V.V. (2015). omparison of barium titanate thin films prepared by inkjet printing and spin coating. Process. Appl. Ceram..

[26] Lau P.H., Takei K., Wang C., Ju Y., Kim J., Yu Z., Takahashi T., Cho G., Javey A. (2013). Fully printed, high performance carbon nanotube thin-film transistors on flexible substrates. Nano Lett..

