# Supplementary material for: Inkjet-Printed Molybdenum Disulfide and Nitrogen-Doped Graphene Active Layer High On/Off Ratio Transistors
Source: Molecules. 2020 Feb 28;25(5):1081. doi: 10.3390/molecules25051081 (PMC7179098; doi:10.3390/molecules25051081)
Supplement: Supplementary file 1 [file molecules-25-01081-s001.pdf]

Inkjet-Printed Molybdenum Disulfide and Nitrogen-Doped Graphene Active Layer High On/Off Ratio Transistors

Mohi Uddin Jewel [1], Mahmuda Akter Monne [2], Bhagyashree Mishra [2], and Maggie Yihong Chen [1,2,*]

[1] Ingram School of Engineering, Texas State University, San Marcos, 78666, Texas, USA;
[2] Materials Science, Engineering, and Commercialization, Texas State University, San Marcos, 78666, Texas, USA;
* Correspondence: Maggie.chen@txstate.edu; Tel.: +1-512-996-8778

Table of Contents:

1. Table of Raman measurements

2. Optical image of TFT

3. Comparison of device current on/off ratios

**Table S1**. Data table for Raman measurements									
Number of printing passes	Observation Number	D Peak Position (cm^{-1})	G Peak Position (cm^{-1})	2D peak position (cm^{-1})	I_D (a.u.)	I_G (a.u.)	I_{2D} (a.u.)	I_D / I_G	I_{2D} / I_G
2	1	1350	1586	2690	346.907	988.156	228.163	0.351	0.230
	2	1347	1586	2682	622.268	1712.24	48.092	0.362	0.028
	3	1352	1587	2698	642.474	1342.88	66.7688	0.478	0.049
	4	1351	1583	2682	341.001	824.549	61.9599	0.413	0.075
5	1	1368	1588	2693	675.994	805.079	118.872	0.839	0.147
	2	1348	1590	2692	1002.75	2428.2	70.0268	0.412	0.028
	3	1339	1580	2666	727.288	2815.1	164.017	0.258	0.058
	4	1348	1588	2708	1269.83	1317.21	113.57	0.964	0.086
10	1	1345	1568	2693	1569.52	2468.49	397.929	0.635	0.161
	2	1345.38	1589	2711	1438.95	1587.01	164.004	0.906	0.103
	3	1344	1583	2686	1420.34	1450.28	247.944	0.979	0.170
	4	1349	1594	2686	1620.22	1729.49	165.427	0.936	0.095
Powder	1	1342	1577	2675	266.177	313.886	59.9369	0.848	0.190
	2	1341	1575	2672	263.555	250.813	50.5023	1.050	0.201
	3	1333	1572	2667	372.381	338.206	70.9321	1.101	0.209
	4	1341	1569	2680	251.053	271.474	74.2074	0.924	0.273

Optical Image of TFT:

An optical image of final device is displayed in **Figure S1.** The top-illumination on the device causes the shadow on the glass substrate background. The source/drain and gate contacts are looking dissimilar due to the different curing temperatures.

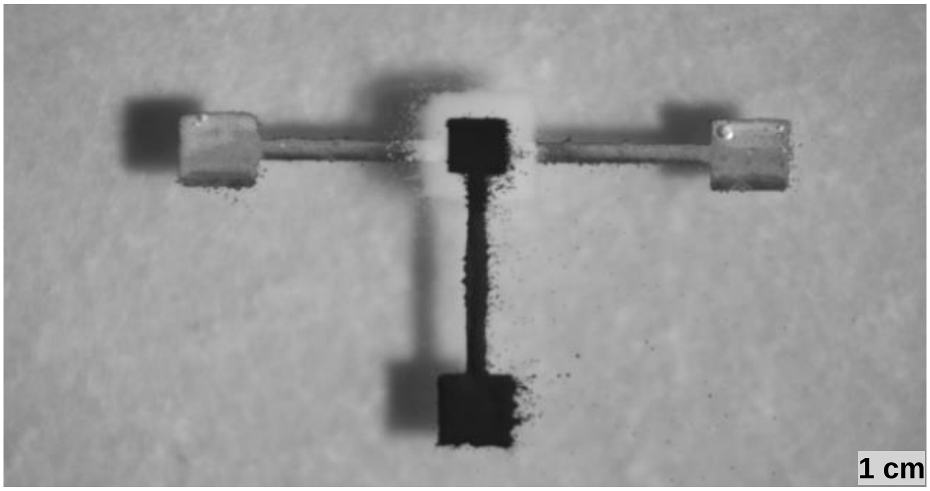

Figure S1. An optical image of final transistor.

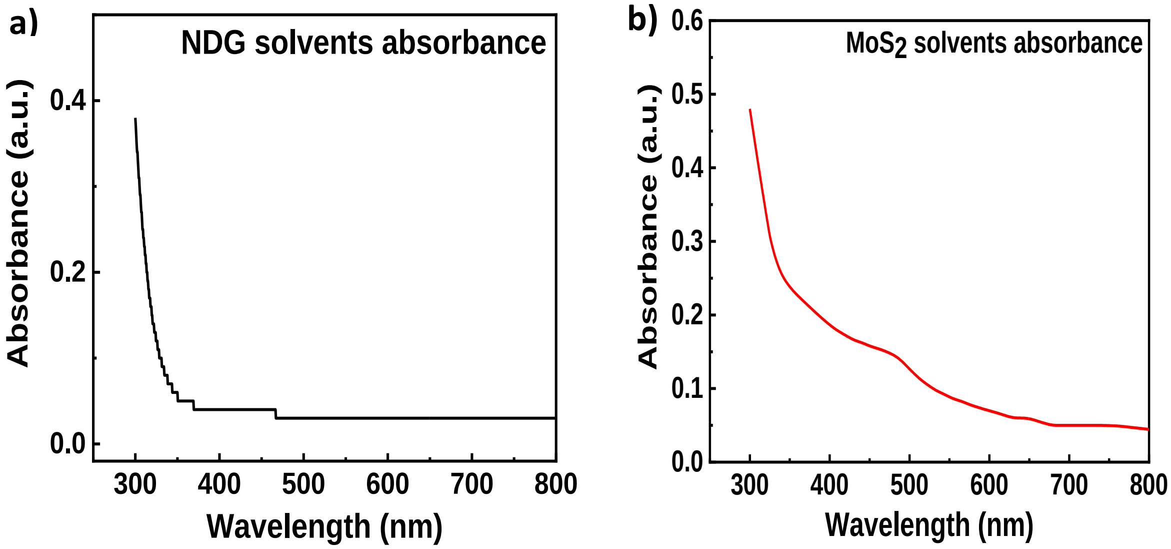

Figure S2. Absorbance spectrum for solvents of a) NDG ink, and b) MoS_2 ink.

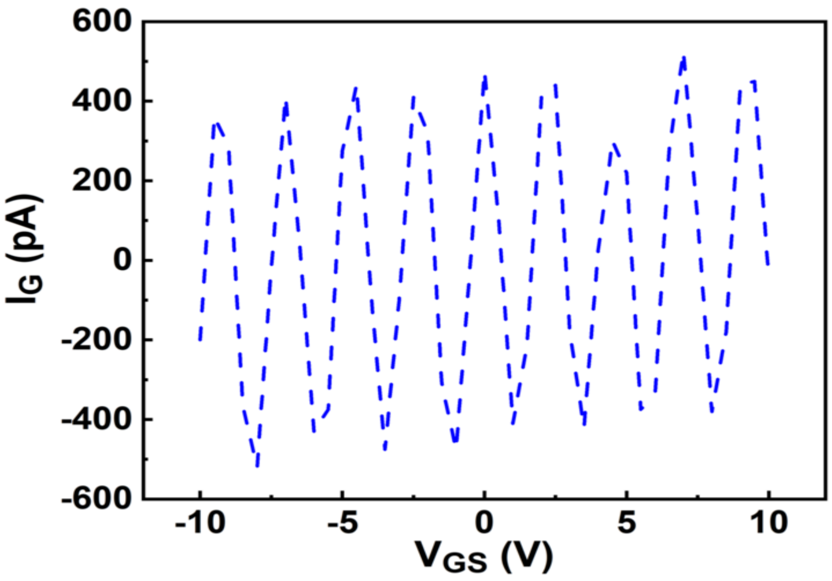

Figure S3. Gate leakage measurement of MoS_2 – NDG transistor.

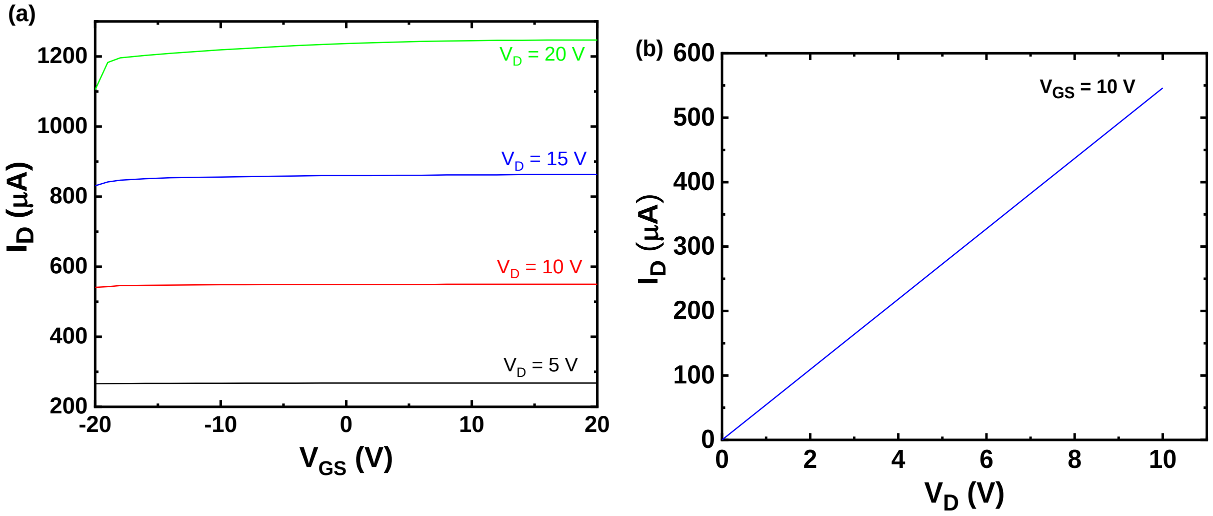

Figure S4. a) Transfer characteristics, and b) output curve of NDG transistor.

Comparison of Current On/Off Ratios:

Table S2 compares the current ratios and synthesis methods of 2D material TFTs. The comparison is also depicted with a bar graph in **Figure S5**. The LPE and IJP stand for liquid phase exfoliation, and inkjet printing respectively. The current on/off ratio of an MoS_2-NDG transistor is very high

compared to the reported IJP and LPE deposited 2D material transistors. For the TFTs of reference 11, they sprayed the dielectric and the measurements were carried out under ultra-high vacuum and low temperature. These devices were not all inkjet printed. 100% inkjet-printed devices do not require photolithography patterning, or surface pretreatment steps, and a complete device can be fabricated with one inkjet printer. In summary, we developed a 2D materials based, 100% inkjet-printed, high current on/off ratio transistor based on 2D materials active or channel layer.

Table S2. Comparison of on/off ratios of 2D materials TFTs

Reference	On/Off Ratio	Material	Deposition	100% inkjet printed
1	10	Graphene	IJP	No
2	2.5	Graphene	IJP	Yes
3	<10	MoS_2	LPE	No
4	3 ~ 6	MoS_2	IJP	No
5	1.2	Graphene	IJP	No
6	3 ~ 4	MoS_2	LPE	No
7	10	MoS_2	IJP	No
8	25	WSe_2	IJP	No
Our work	1200	MoS_2-NDG	IJP	Yes

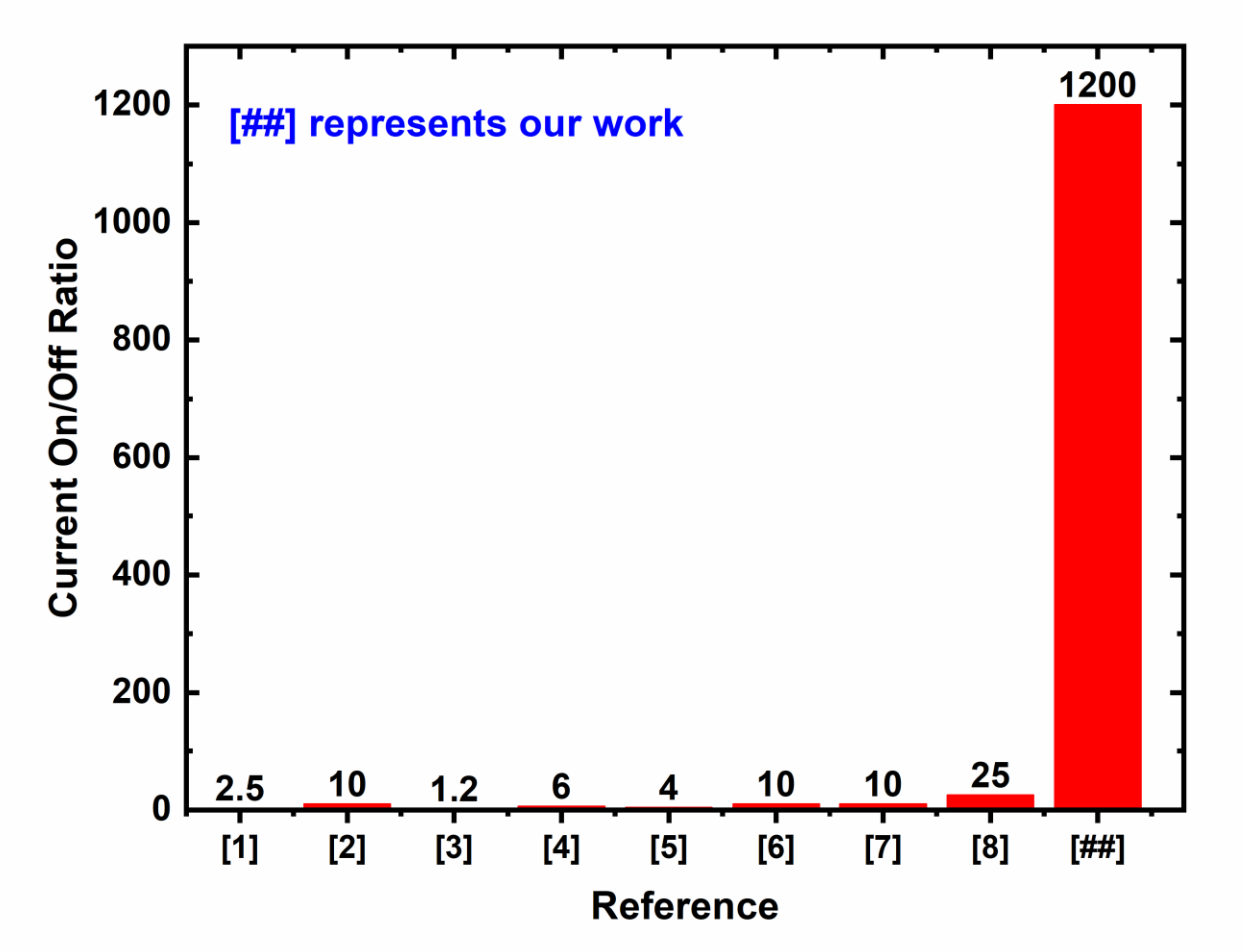

Figure S5. Comparison of previously reported current on/off ratios.

References

1. Torrisi, F.; Hasan, T.; Wu, W.; Sun, Z.; Lombardo, A.; Kulmala, T. S.; Hsieh, G.-W.; Jung, S.; Bonaccorso, F.; Paul, P. J. Inkjet-printed graphene electronics. *ACS Nano* **2012**, 6, 2992–3006. doi:10.1021/nn2044609

2. Carey, T.; Cacovich, S.; Divitini, G.; Ren, J.; Mansouri, A.; Kim, J. M.; Wang, C.; Ducati, C.; Sordan, R.; Torrisi, F. Fully inkjet-printed two-dimensional material field-effect heterojunctions for wearable and textile electronics. *Nat. Commun.* **2017**, 8, 1202. doi:10.1038/s41467-017-01210-2

3. Coleman, J. N.; Lotya, M.; O'Neill, A.; Bergin, S. D.; King, P. J.; Khan, U.; Young, K.; Gaucher, A.; De, S.; Smith, R. J. Two-dimensional nanosheets produced by liquid exfoliation of layered materials. *Science* **2011**, 331, 568–571. doi:10.1126/science.1194975

4. Li, J.; Naiini, M. M.; Vaziri, S.; Lemme, M. C.; Östling, M. Inkjet printing of MoS_2. *Adv. Funct. Mater.* **2014**, *24*, 6524–6531. doi:10.1002/adfm.201400984

5. Li, J.; Ye, F.; Vaziri, S.; Muhammed, M.; Lemme, M. C.; Östling, M. Efficient inkjet printing of graphene. *Adv. Mater.* **2013**, *25*, 3985–3992. doi:10.1002/adma.201300361

6. Lee, K., Kim, H.-Y., Lotya, M., Coleman, J. N., Kim, G.-T., & Duesberg, G. S. Electrical Characteristics of Molybdenum Disulfide Flakes Produced by Liquid Exfoliation. *Adv. Mater.* **2011**, 23, 4178–4182. doi:10.1002/adma.201101013

7. He, Q.; Zeng, Z.; Yin, Z.; Li, H.; Wu, S.; Huang, X.; Zhang, H. Fabrication of flexible MoS_2 thin-film transistor arrays for practical gas-sensing applications. *Small* **2012**, *8*, 2994–2999. doi:10.1002/smll.201201224

8. Kelly, A. G.; Hallam, T.; Backes, C.; Harvey, A.; Esmaeily, A. S.; Godwin, I.; Coelho, J.; Nicolosi, V.; Lauth, J.; Kulkarni, A. All-printed thin-film transistors from networks of liquid-exfoliated nanosheets. *Science* **2017**, *356*, 69–73. doi:10.1126/science.aal4062
